# More evidence is needed on the effectiveness of kinesio taping for treating lateral humeral epicondylitis

**DOI:** 10.1097/JS9.0000000000002421

**Published:** 2025-04-22

**Authors:** Wen-Hsuan Hou, Po-Han Tsou, Jeng-Cheng Wu

**Affiliations:** a Department of Physical Medicine and Rehabilitation, Taipei Medical University Hospital, Taipei, Taiwan; b Department of Physical Medicine and Rehabilitation, School of Medicine, College of Medicine, Taipei Medical University, Taipei, Taiwan; c Graduate Institute of Clinical Medicine, College of Medicine, Taipei Medical University, Taipei, Taiwan; d School of Gerontology and Long-Term Care, College of Nursing, Taipei Medical University, Taipei, Taiwan; e Cochrane Taiwan, Taipei Medical University, Taipei, Taiwan; f Department of Primary Care Medicine, Wan Fang Hospital, Taipei Medical University, Taipei, Taiwan; g Department of Medical Education, Taipei Medical University Hospital, Taipei, Taiwan; h Department of Urology, Taipei Medical University Hospital, Taipei, Taiwan; i Department of Education and Humanities in Medicine, School of Medicine, College of Medicine, Taipei Medical University, Taipei, Taiwan; j Department of Urology, School of Medicine, College of Medicine, Taipei Medical University, Taipei, Taiwan; k TMU Research Center of Urology and Kidney (TMU-RCUK), Taipei Medical University, Taipei, Taiwan; l Department of Health Promotion and Health Education, College of Education, National Taiwan Normal University, Taipei, Taiwan

*Dear Editor,*

We read with interest of a comprehensive synthesis about effects of kinesio taping (KT) for lateral humeral epicondylitis (LHE) by Zhu *et al* in the *International Journal of Surgery* in 2025^[^1^]^. The study extends the previous study by Zhong *et al* in the *International Journal of Surgery* in 2020^[^2^]^, which provides a larger sample size and valuable contribution to the ongoing discussion surrounding non-surgical treatments for this common musculoskeletal condition based on relevant randomized controlled trials (RCTs), and shows significant reductions in pain and improvements in function. However, we have some concerns about the interpretation of the conclusion that “…*KT can effectively and safely relieve pain during activity and improve LHE-related function, especially in the long term…,*” which is based on a very small sample size. This correspondence aims to shed light on the results of Zhu *et al*, reflecting on the clarity and conclusiveness of the evidence. Brok *et al* have been proposed the caution of type I errors should be addressed because meta-analyses repetitively testing accrued data and multiple outcomes, and suggested trial sequential analysis (TSA) is an approach for meta-analyses not reaching the required sample size^[^3^]^. Thus, by examining the current landscape of research, we can better understand the evidence on the role of KT in managing LHE.

**HIGHLIGHTS**

- Re-analysis indicated that the effect of Kinesio taping for tennis elbow remains inconclusive, after adjusting for sample size limitations.
- The presence of small sample sizes in studies increase the risk of type I errors, warranting caution in interpretation.
- Additional large-scale, well-designed randomized trials are recommended to confirm long-term effectiveness of Kinesio taping for tennis elbow management.

According to the meta-analysis that reported the largest effect size in the research conducted by Zhu *et al*, we gathered data on the total score of the Patient-Rated Tennis Elbow Evaluation (PRTEE) at the six-month follow-up. Our methodology mirrored the approach in the meta-analysis by Zhu et al., employing standardized mean difference (SMD) within a random-effects model using the DerSimonian-Laird method, with results reported with 95% confidence intervals (CI). Then, we further used sequential methods, setting a type I error of 0.05, power of 0.80, and both a moderate effect size (SMD = 0.5) and observed effect. While the TSA program (https://ctu.dk/tsa/) does not accommodate analyses that utilize SMD as the effect size, we adopted the methodology outlined by Kang *et al*^[^4^]^ and Fang *et al*^[^5^]^ using R software (R-4.4.0, https://cran.rstudio.com/) within Rstudio.

We not only reproduced the results obtained by Zhu *et al*, but also further identified inconclusive evidence regarding the effectiveness of KT on the PRTEE among patients with LHE. As Zhu et al. reported, their analysis included three studies with a total of 81 patients in the KT group and 81 patients in the control group. We replicated their finding of a significant reduction in the PRTEE score for the KT group compared to the control group (SMD: −2.52; 95% CI: −3.55 to −1.49; Fig. 1A). However, in our supplementary analysis, the acquired information size (AIS) of 162 patients did not exceed the required information size (RIS) of 35 336 or 1392, regardless of whether we used the commonly accepted medium effect size of 0.5 or the observed effect size of 2.52 (Fig. 1B and 1C). The cumulative *z*-score of −4.794 for the combined results was well outside the *α*-spending monitoring limits (<−10 or >10). The 95% CIs, adjusted for the spending boundaries, spanned from −5.377 to 0.332. This indicates that, after adjusting for type I error, KT did not lead to a significant reduction in the PRTEE score when compared to the control group.

**Figure 1.**
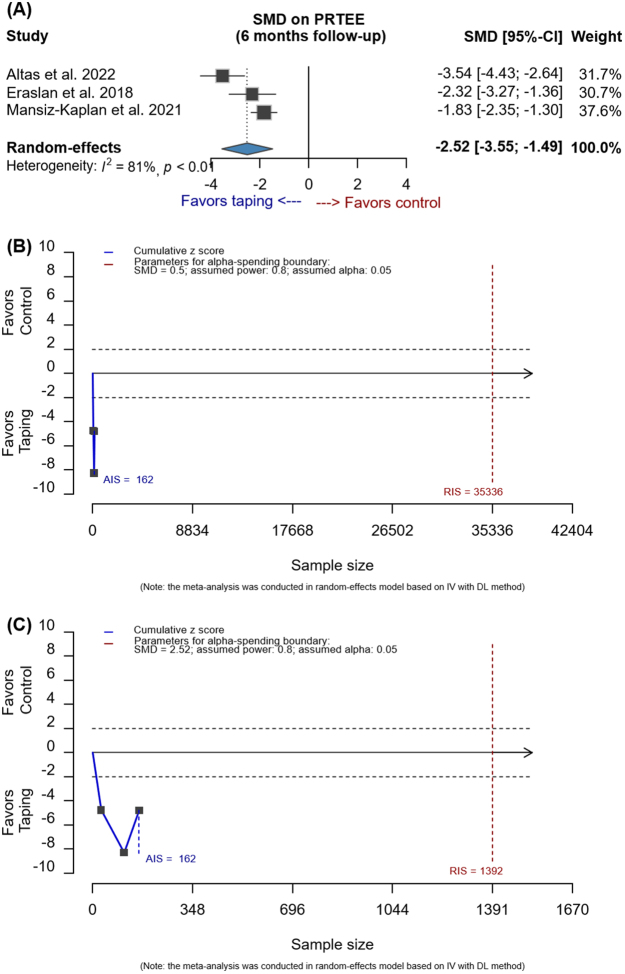
Result of the patient-rated tennis elbow evaluation score in (A) meta-analysis, (B) sequential analysis based on medium effect size, and (C) sequential analysis based on observed effect. AIS, acquired information size; CI, confidence interval; DL, DerSimonian-Laird’s between-study variance estimator; IV, inverse-variance method; PRTEE, patient-rated tennis elbow evaluation; RIS, required information size; SMD, standardized mean difference

In conclusion, while meta-analysis by Zhu *et al* initially suggested a significant long-term benefit of KT for LHE, our re-analysis, incorporating sequential methods to account for potential type I errors and insufficient sample size, reveals a different picture. Despite replicating their reported significant reduction in PRTEE scores, the sequential analysis demonstrated that the accumulated evidence, based on the limited number of studies and patients, was inconclusive. The required information size was far from being reached, and the adjusted confidence intervals indicated a lack of significant effect of KT. Therefore, the claim that KT effectively and safely relieves pain and improves LHE-related function, particularly in the long term, should be cautiously interpreted due to insufficient robust evidence. Further well-designed, large-scale randomized controlled trials are encouraged to provide a more definitive understanding of KT’s efficacy in managing LHE.

## Data Availability

All data generated or analyzed during this study are included in this published article.
